# Multi‐assay characterization of human iPSC derived microglia‐like cells in response to recombinant antibodies and immunogenic stimuli

**DOI:** 10.1002/alz70855_103179

**Published:** 2025-12-23

**Authors:** Joshua Kulas, Angela K Haskell, William Carter, Jacob Smiley, Raven Hinkel, Mustapha Moussaif, June Javens‐Wolfe, Shaoyou Chu, Timothy I. Richardson, Abdul Syed

**Affiliations:** ^1^ Indiana Biosciences Research Institute (IBRI), Indianapolis, IN, USA; ^2^ Indiana Biosciences Research Institute, Indianapolis, IN, USA; ^3^ Indiana University School of Medicine, Indianapolis, IN, USA

## Abstract

**Background:**

iPSC technology has enabled the interrogation of human microglia‐like cells *in vitro*. In this study, we differentiated iPSC derived microglia‐like cells (iMG) using a newly generated cell line. We perform a number of functional and biochemical evaluations of the cell culture model and treat the cells with a variety of immunogenic stimuli including myelin debris and Aβ oligomers. Finally, we investigate antiamyloid and TREM2 targeting antibodies for their impacts on iMG cell biology.

**Method:**

The IBRI 104.G iPSC line was generated from donor cells using episomal reprogramming vectors. iMG were differentiated on matrigel coated plates using previously published methods. Myelin debris was harvested from mouse brains, while Aβ oligomers were generated from recombinant peptides. TREM2 and Aβ antibodies were produced by transfection of CHO cells using heavy and light chain plasmids. High content fluorescent imaging was utilized to track microglia cell morphology and phagocytosis over time.

**Result:**

The IBRI 104.G iPSC line was found to express a number of pluripotent cell markers and transcription factors. Characterization of iMG revealed several microglia cell markers including PU.1, P2RY12R, TMEM119 and TREM2. iMG displayed prominent ramified morphology under control conditions and rapidly became ameboid upon stimulation with inflammatory molecules. iMG rapidly phagocytosed myelin debris and amyloid beta, and downregulated homeostatic markers upon stimulation. Treatment of iMG with monoclonal TREM2 antibodies reduced the rate of myelin phagocytosis, while Aβ antibodies enabled the rapid uptake of amyloid resulting in increased methoxy‐X04 and lysotracker fluorescence.

**Conclusion:**

The IBRI 104.G iPSC line readily differentiates into highly ramified microglia‐like cells that exhibit many properties of human microglia. iMG may serve as a valuable model system for examining tool molecules *in vitro* which seek to modulate neuroinflammation by targeting brain myeloid cells.

